# Left Subclavian Artery Pseudoaneurysm after a Traffic Accident: A Case Report

**DOI:** 10.1155/2011/451819

**Published:** 2011-07-14

**Authors:** J. Enamorado-Enamorado, J. J. Egea-Guerrero, J. Revuelto-Rey, E. Gordillo-Escobar, C. Herrera-Melero

**Affiliations:** Critical Care Unit, Virgen del Rocío University Hospital, 41013 Seville, Spain

## Abstract

The left subclavian artery pseudoaneurysm is a rare entity with few cases reported in the literature. Most injuries were related to iatrogenic manipulation with catheters for canalization of central lines. In rare cases, this injury has been described secondary to a blunt trauma. We present an unusual case of pseudoaneurysm that includes the origin of left subclavian artery in the context of severe multiple injuries after a traffic accident. There were not clavicular or rib fractures, or another type of chest trauma to justify such a vascular injury. Once the injuries that were life threatening for the patient were stabilized, it proceeded to the treatment of the pseudoaneurysm by placing an endovascular prosthesis successfully with a favorable clinical evolution.

## 1. Case Report

**Figure 1:**
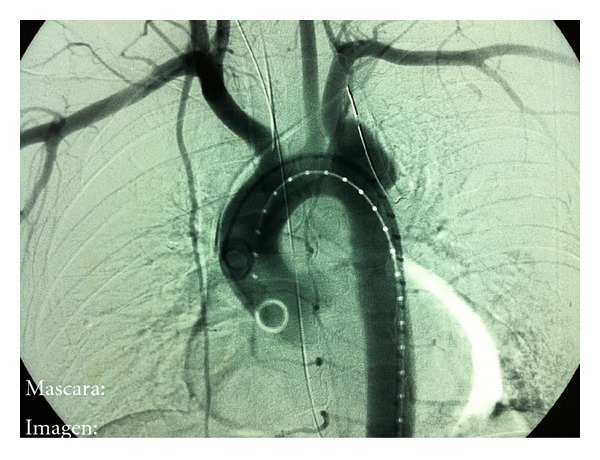
Arteriography where it is observed the pseudoaneurysm of the left subclavian artery.

**Figure 2:**
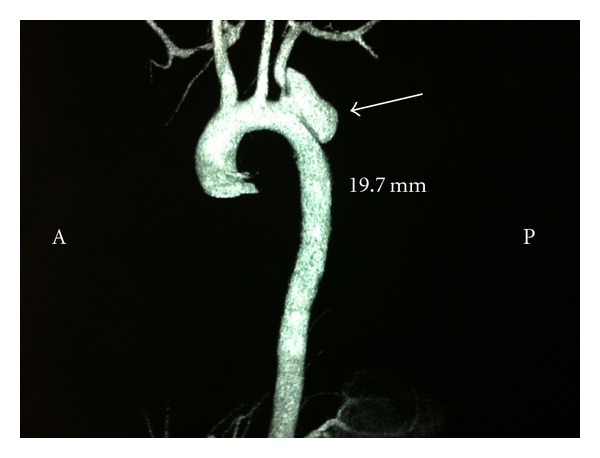
Angio-CT where we can appreciate the pseudoaneurysm (white arrow) which includes the origin of the left subclavian artery without affecting the left carotid artery.

This is a 24-year-old man with no relevant history, which has a polytrauma by collision with another vehicle. It was attended by the emergency service in a situation of hemodynamic instability and acute respiratory failure, needing an endotracheal intubation, and he was moved urgently to the hospital. Upon admission to the emergency room and after stabilization, in the initial radiographs it was observed an acetabular fracture and dislocated right hip without a demonstrable clavicular, rib, or sternum fractures. In a later study of Angio-CT it was observed a pseudoaneurysm secondary to traumatic rupture that included the origin of the left subclavian artery (see Figures 1 and 2). The rupture appears to extend a few millimeters from the origin of the left carotid artery. The rest of the thoracic aorta and supraaortic vessels were normal. There was no clinical signs (no pulsatile mass, localized pain, or murmurs), associated with this finding. During his hospital stay, the patient firstly presented multiple septic and respiratory complications. In a second stage the placement of a stent at the aortic arch and an Amplatzer device in the left subclavian artery, proximal to the exit of the vertebral artery, for the treatment of the pseudoaneurysm were carried out successfully. The procedure was done without complications and with good evolution of the patient remaining asymptomatic at the time of discharge.

## 2. Discussion

Blunt trauma of the brachiocephalic vessels is relatively rare [1]. The real incidence of lesions in the supraaortic vessels secondary to a blunt trauma is difficult to determine, being underestimated because most of the patients suddenly die and are rarely included in clinical series of vascular lesions [2]. In 1962, Binet et al. [3] suggested the physiopathological mechanisms by which it produces this type of injury: “The crushing forces acting in anteroposterior direction and shorten the distance between the sternum and the spine, while the heart and aorta are then diverted to the left, and curvature of the aortic arch is accentuated by increasing the tension in the portion convex, where the brachiocephalic trunk has its origin. Furthermore, the patient adopts an attitude of defense against facial trauma and hyperextended cervical spine rotating the head to one side and thus tightening the carotid, which causes tear at the junction of the aortic arch and the carotid artery.” This would explain the vascular injury without rib or clavicular lesions. The subclavian artery lesions are rare and potentially catastrophic representing less than 5% of all vascular lesions. The vast majority of the subclavian artery lesions are the result from penetrating trauma, and approximately 25% of these injuries are caused by blunt trauma. It has been described an overall mortality rate around 60% in patients that did not make it into the hospital because they died during transportation or at the crash site. The hospital mortality rate is about 5 to 30% in the patients that survived the triggering event. The leading causes of death in the subclavian artery lesions are massive bleeding and traumatic brain injury [4]. This type of injury is not well characterized. The delay in diagnosis and complications associated with surgical repair influence patient outcomes.

Within the subclavian artery lesions are the pseudoaneurysms, defined as a contained rupture of the arterial wall, in which true blood collection without walls, is still in contact with the artery through a channel. The most frequent traumatic pseudoaneurysms are in the common femoral artery; the majority are secondary to arterial catheterization, infections, surgical procedures, and/or radiology interventions, and in a very few cases have been described the injury of the subclavian artery secondary to a blunt trauma. Early diagnosis through imaging tests such as Angio-CT or arteriography can, in most of the cases, save the life of these patients [5]. Previously, the only available treatment for subclavian artery pseudoaneurysm was surgery. This intervention involved either resection or exclusion of the aneurysm by direct reconstruction of the vessel or by an extra-anatomic bypass. This procedure was complicated and often required an intrathoracic sternotomy. Recently, less invasive procedures have been developed for exclusion of the pseudoaneurysm. The first reported case of endovascular repair of a subclavian artery was in an attempt to prevent massive bleeding by iatrogenic perforation. This type of approach is currently being used to exclude pseudoaneurysms. However, experience in subclavian arteries remains limited because of its relative infrequency [6].
